# European studies to evaluate COVID-19 vaccine effectiveness in HCWs: results from Italian hospitals: Antonella Agodi

**DOI:** 10.1093/eurpub/ckac130.021

**Published:** 2022-10-25

**Authors:** A Agodi, V Rapisarda, P Bonfanti, M Rossi, K De Gaetano Donati, R Murri, M Barchitta

**Affiliations:** 1 AOU Policlinico G. Rodolico - San Marco, University of Catania, Catania, Italy; 2 University of Catania, Catania, Italy; 3 Department MEDCLIN, University of Catania, Catania, Italy; 4 San Gerardo Hospital, Hospital, Monza, Italy; 5 University of Milano-Bicocca, University, Milan, Italy; 6 Fondazione PU Agostino Gemelli, IRCCS, Rome, Italy

## Abstract

**Background:**

Evaluating COVID-19 vaccine coverage and risk factors is useful to develop public health strategies against COVID-19 pandemic. In the framework of two studies commissioned by the European Centre for Disease Prevention and Control (ECDC) and coordinated by Epiconcept, France, we reported findings about incidence and seroprevalence among healthcare workers (HCWs) enrolled from three Italian hospitals.

**Methods:**

From July 2021 to date, the AOUP “G. Rodolico-San Marco” (Catania), the San Gerardo Hospital (Monza) and the Policlinico Gemelli (Rome) participated in the ECDC study to measure COVID-19 vaccine effectiveness. Catania and Rome also participated to the ECDC study of nosocomial transmission. HCWs were asked to complete a weekly questionnaire to report changes in health status and professional/personal exposures. At recruitment, a nasopharyngeal swab for RT-PCR and a blood sample for serology test were collected. Moreover, HCWs were followed-up with a weekly or bimonthly nasopharyngeal or saliva swabs. Blood samples were collected every one or two months.

**Results:**

A total of 226 HCWs was enrolled from Catania, 330 from Rome and 132 from Monza in the COVID-19 vaccine effectiveness study. As of February 2022, PCR tests performed were 2270 in Catania, 5475 in Rome and 891 in Monza sites. Moreover, the serological tests performed were 845 in Catania, 760 in Rome and 395 in Monza sites. A total of 6 SARS-CoV-2 infections were identified in Catania, 34 in Rome and 4 in Monza sites. Interestingly, the study of nosocomial transmission reported the highest incidence rate in Catania (4 per 10,000 person-day), while 0.7 per 10,000 person-day in Rome. During the study period seroprevalence declined by 17% among HWCs enrolled in Catania.

**Conclusions:**

Our findings revealed low number of COVID-19 infections, with high COVID-19 vaccine coverage among HCWs. However, further analyses are needed to provide more robust estimates of vaccine effectiveness.

**Key messages:**

- Monitoring risk factors is useful to understand the pandemic scenario among high-risk groups.
- A larger sample size and follow-up will improve public health vaccination strategies against COVID-19.

